# A systematic review of the association between delayed appropriate therapy and mortality among patients hospitalized with infections due to *Klebsiella pneumoniae or Escherichia coli:* how long is too long?

**DOI:** 10.1186/s12879-018-3524-8

**Published:** 2018-12-05

**Authors:** Thomas P. Lodise, Qi Zhao, Kyle Fahrbach, Patrick J. Gillard, Amber Martin

**Affiliations:** 1 Department of Pharmacy Practice, Albany College of Pharmacy and Health Sciences, Albany, NY USA; 2 Global Health Economics and Outcomes Research, Allergan plc, Madison, NJ USA; 3 Meta Research, Evidera, Bethesda, MD USA; 4 Global Health Economics and Outcomes Research, Allergan plc, 2525 Dupont Drive, Irvine, CA 92612 USA

**Keywords:** *Escherichia coli*, Gram-negative infection, *Klebsiella pneumoniae*, Mortality, Delayed treatment

## Abstract

**Background:**

Temporal relationships between the time to appropriate antibiotic therapy and outcomes are not well described.

**Methods:**

A systematic literature review and meta-analysis was performed to examine this relationship in patients hospitalized with *Klebsiella pneumoniae* or *Escherichia coli* infections.

**Results:**

Twenty identified studies contained data for patients who received delayed appropriate therapy (DAT) versus appropriate antibiotic therapy for these pathogens. Of the 20 included studies, the majority (19/20) focused on patients with bloodstream infections, and only 1 study evaluated patients with pneumonia. When all DAT results were combined (any delay > 24 h from culture collection or any delay after culture and susceptibility reporting [C& SR]), there was an increased risk of mortality (odds ratio [OR], 1.60 [95% CI, 1.25–2.50]). The risk of mortality was greater when DAT > 48 h from culture collection or DAT > C&SR results were combined (OR, 1.76 [95% CI, 1.27–2.44]).

**Conclusions:**

Our findings suggest there is a need to shift current treatment practices away from antibiotic escalation strategies that contribute to delayed appropriate therapy and toward early, relatively aggressive and comprehensive, antibiotic therapy, especially among patients with bloodstream infections due to *K. pneumoniae or E. coli*.

**Electronic supplementary material:**

The online version of this article (10.1186/s12879-018-3524-8) contains supplementary material, which is available to authorized users.

## Background

Recent data from the Study for Monitoring Antimicrobial Resistance Trends program [1] found that during 2011 the global rates of extended-spectrum beta lactamase (ESBL)-containing *Escherichia coli* from intra-abdominal infections ranged from 6% (North America) to 38% (Asia). During the preceding decade, rates for urinary tract infections (UTIs) caused by ESBL *E. coli* and *Klebsiella pneumoniae* increased from 3.3 to 8.0% and from 9.1 to 18.6%, respectively, across 217 hospitals in the United States [2]. Results from the Healthcare Cost and Utilization Project Nationwide Inpatient Sample database [2] show that the number of complicated (ie, hospitalized) UTIs caused by carbapenem-resistant *Enterobacteriaceae* (CRE) in the United States increased from 0% in 2000 to 2.3% in 2009. The clinical burden of these resistant infections is substantial: ESBL-producing *Enterobacteriaceae* and CRE account for an estimated 1700 and 610 deaths annually, respectively, in the United States [3].

Reasons for concern regarding the rising rates of resistance are many. In more than 25% of patients with resistant Gram-negative infections, initial empirical antibiotic therapy has no microbiological activity against the pathogen causing the infection [4, 5]. Patients who receive delayed appropriate therapy (DAT) may have a 1.5- to 2-fold increase in morbidity and mortality relative to those who receive early appropriate therapy [6].

A second reason for concern is that resistance limits treatment options. For ESBLs, clinicians are often left using last-line agents, such as carbapenems, empirically. Clinicians have had to rely on older, often toxic agents (eg, colistin) with limited efficacy data to treat infections caused by CRE [7, 8].

Although the deleterious outcomes associated with DAT are well described, the definitive time delay associated with worse outcomes has not been well quantified. There is no standardized definition of DAT; different definitions are used throughout the literature, with DAT variously defined as nonreceipt of microbiologically active treatment within 24, 48, or 72 h of culture collection or at the time of culture and susceptibility reporting (C& SR). Thus, the question remains: how long a delay is too long? The intent of this study was to examine the association between delay in appropriate therapy and mortality in patients with infections due to *K. pneumoniae or E. coli*.

## Methods

This review was conducted in accordance with the Preferred Reporting Items for Systematic Reviews and Meta-Analyses (PRISMA) guidelines.

### Search strategy

Systematic searches were conducted in the MEDLINE (via PubMed), EMBASE, and Cochrane literature databases. Because the terms “inappropriate therapy” and “delayed appropriate therapy” are used interchangeably in the literature, both terms were paired with terms for Gram-negative pathogens and infection types of interest to identify English-language studies published between January 1, 2004, and June 13, 2014. Complete details of the search algorithms are provided in Additional file 1.

### Inclusion and exclusion criteria

All identified abstracts were manually reviewed against the prespecified inclusion/exclusion criteria considering participants, interventions, comparisons, outcomes, study design, and time period (PICOS-T) elements. Included studies evaluated adults hospitalized with Gram-negative infections (ie, pneumonia, bloodstream infections, complicated UTIs, and complicated intra-abdominal infections) caused by *K. pneumoniae* or *E. coli*. Studies were required to have extractable outcomes data regarding DAT, which was defined as nonreceipt of microbiologically active treatment within 24, 48, or 72 h of culture collection (DAT > 24, > 48, or > 72 h) or at the time of C& SR (DAT > C& SR). Studies that involved pediatric patients; used treatments other than antibiotics; were animal, in vitro, or genetic; were narrative publications, nonsystematic reviews, case studies, case reports, or editorials; or were comparative studies with < 10 patients per treatment group or single-arm studies with fewer than 20 patients were excluded. Also excluded were studies reporting < 5% of patients receiving DAT > C& SR compared with those who received appropriate antibiotics.

### Data extraction and statistical analysis

Extraction of the included studies was performed using a Microsoft Excel® extraction template. Meta-analysis was conducted using R version 2.15.2 [9]. Study-level data, patient characteristics, treatment details, and mortality were captured from full-text articles by a single investigator. All extracted data were validated by a second investigator to confirm the completeness and accuracy of extraction. The validated data were eligible for inclusion in statistical analysis. Random-effects meta-analyses of odds ratios (ORs) for mortality were conducted when data permitted for the following DAT definitions by *K. pneumoniae* and *E. coli* combined and by each organism individually:

- All DAT (includes studies with DAT > 24, > 48, or > 72 h from the time of culture collection and results from studies with DAT > C& SR)
- DAT > 24 h and DAT > C& SR, combined
- DAT > 48 h and DAT > C& SR, combined
  o This approximates the typical 48- to 72-h time frame for reporting of culture and susceptibility data

- Additional DAT categories
  o DAT > 24 h
  o DAT > 48 h
  o DAT > 72 h
  o DAT > C& SR

For clarity, studies that include a DAT > 24 h may include delays in starting appropriate therapy of > 48 h; studies that include a DAT of > 48 h may include delays in starting appropriate therapy of > 72 h but will not include delays in starting therapy of < 48 h.

The random-effects method considers any variation between studies as a random variable, enabling the analysis to account for any heterogeneity observed. Heterogeneity was quantified using classical meta-analysis methods (ie, Cochran Q and the I^2^ measure). Heterogeneity is defined as differences between study results beyond what would be expected from sampling error (ie, τ^2^ > 0) [10]. It may stem from several sources, including differences across studies in patient inclusion and exclusion criteria, differences in accuracy of laboratory measurement, and differences in reporting conventions (eg, group means at baseline vs sensitivity/specificity).

## Results

### Systematic literature search

The literature searches yielded 1844 unique articles after results from each database were merged and duplicate citations were removed. Of these, 1550 studies were rejected during abstract screening because they did not meet inclusion criteria.

**Fig. 1:**
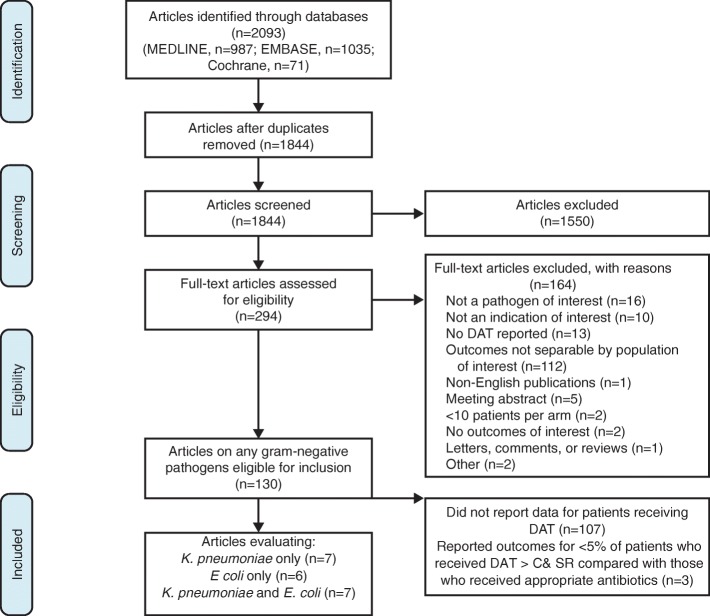
Study attrition

Full-text articles of the remaining 294 citations were retrieved for further examination, during which 164 articles were rejected, most commonly because they did not report mortality for the population of interest (ie, patients infected with *Klebsiella* spp. or *E. coli*; *n* = 112). Of the remaining 130 studies, 110 were excluded because they either did not report outcomes data for patients receiving DAT (*n* = 107) or they reported outcomes for patients who received DAT > C& SR, the proportion of whom was < 5% of those patients who received appropriate antibiotics in the same study (*n* = 3). An overview of the reasons for study attrition is shown in Fig. 1. Of the 20 included studies, the majority (19/20) focused on patients with bloodstream infections, and only 1 study evaluated patients with pneumonia [11]. Table 1 [11–30] summarizes the mortality results for the 20 included studies.

**Table 1:** Studies Reporting Overall Mortality Data for Delayed Appropriate Therapy of Infections Due to *Klebsiella pneumoniae* or *Escherichia coli*

Author, Year	Time Point for DAT, h	Pathogen	Resistance Status	Therapy Type	Infection Type	n/N	Mortality Rate, %
Abhilash et al., 2010 [12]	48	*K. pneumoniae* or *E. coli*	73.3% ESBL producing	Appropriate	Bacteremia; primary sources: UTI, intra-abdominal, pneumonia	17/74	23.0
				DAT		16/57	28.1
Anderson et al., 2006 [13]	72	*K. pneumoniae*	7.7% CAZ-R (of which 85% ESBL producers)	Appropriate	Bacteremia	12/38	31.6
				DAT		14/22	63.6
Daikos et al., 2009 [11]	NR	*K. pneumoniae*	41.4% VPKP (of which 20.9% CRE)	Appropriate	Bacteremia	23/137	16.8
				DAT		8/25	32.0
Du et al., 2002 [14]	NR	*K. pneumoniae* or *E. coli*	27% ESBL producers	Appropriate	Bacteremia	14/71	19.7
				DAT		7/14	50.0
Gransden et al., 1990 [15]	48	*E. coli*	NR	Appropriate	Bacteremia; primary source: UTI, other^a^	109/659	16.5
				DAT		36/132	27.3
Jung et al., 2012 [16]	NR	*K. pneumoniae*	3.3% ESBL producers	Appropriate	Bacteremia; primary source: UTI, other^b^	82/500	16.4
				DAT		15/54	27.8
Kang et al., 2004 [17]	24	*K. pneumoniae*	100% ESBL producers (study-selected)	Appropriate	Bacteremia; primary source: pancreatobiliary tract, liver, pneumonia, UTI, peritonitis	7/33	21.2
				DAT		3/14	21.4
	24	*E. coli*		Appropriate		2/25	8.0
				DAT		4/23	17.4
Kang et al., 2013 [18]	72	*E. coli*	100% ESBL producers (study-selected)	Appropriate	Bacteremia; primary source: bacteremia, UTI, biliary tract, intra-abdominal, pneumonia	3/28	10.7
				DAT		7/64	10.9
Kim et al., 2002 [19]	72	*K. pneumoniae*	27.2% ESBL producers	Appropriate	Bacteremia; primary source: bacteremia, biliary, pneumonia, UTI, liver, peritonitis	5/19	26.3
				DAT		5/24	20.8
Lee et al., 2012 [30]	NR	*K. pneumoniae* or *E. coli*	100% ESBL producers (study-selected)	Appropriate	Bacteremia; primary source: pneumonia, urosepsis, other^c^	25/230	10.9
				DAT		8/21	38.1
Metan et al., 2005 [20]	NR	*E. coli*	100% ESBL producers (study-selected)	Appropriate	Bacteremia; primary source: pneumonia, UTI, pancreaticobiliary tract, post-surgical wound, cellulitis, abdominal abscess	12/45	26.7
				DAT		2/8	25.0
Micek et al., 2010 [21]	24	*E. coli*	3% ESBL producers	Appropriate	Bacteremia; primary source: lungs, UTI, central venous catheter, intra-abdominal	60/188	31.9
				DAT		11/37	29.7
	24	*K. pneumoniae*	8.5% ESBL producers	Appropriate		47/129	36.4
				DAT		15/30	50.0
Ortega et al., 2011 [22]	NR	*K. pneumoniae*	12% CTX-R	Appropriate	Bacteremia; primary source: UTI, biliary, catheter-related, pneumonia, abdomen, skin and soft tissue	82/824	10.0
				DAT		14/86	16.3
Park et al., 2011 [23]	24	*E. coli*	100% ESBL producers (study-selected)	Appropriate	Bacteremia; primary source: UTI, intra-abdominal; hepatobiliary; skin and soft tissue	10/108	9.3
				DAT		7/42	16.7
Rodriguez-Bano et al., 2010 [24]	24	*E. coli*	100% ESBL producers (study-selected)	Appropriate	Bacteremia; primary source: UTI, intra-abdominal, respiratory tract	16/53	30.2
				DAT		13/43	30.2
Schiappa et al., 1996 [25]	72	*K. pneumoniae* or *E. coli*	100% CAZ-R (study-selected)	Appropriate	Bacteremia	1/19	5.3
				DAT		5/12	41.7
Tuon et al., 2011 [26]	48	*K. pneumoniae*	60% ESBL producers	Appropriate	Bacteremia	28/55	50.9
				DAT		20/49	40.8
Wang et al., 2011 [27]	NR	*K. pneumoniae* or *E. coli*	100% ESBL producers (study-selected)	Appropriate	Bacteremia; primary source: pneumonia, urosepsis, intra-abdominal, bacteremia, vascular catheter-related, skin and soft tissue	12/50	24.0
				DAT		15/63	23.8
Wu et al., 2012 [28]	48	*E. coli*	100% ESBL producers (study-selected)	Appropriate	Bacteremia; primary source: urinary tract, intra-abdominal, bacteremia	4/24	16.7
				DAT		9/38	23.4
Zarkotou et al., 2011 [29]	24	*K. pneumonia*	100% KPC producers (study-selected)	Appropriate	Bacteremia; primary source: central venous catheter, respiratory tract, UTI, skin or soft tissue, central nervous system	5/14	35.7
				DAT		13/39	33.3

*CAZ-R* ceftazidime-resistant, *CRE* carbapenem-resistant Enterobacteriaceae, *CTX-R* cefotaxime resistant, *DAT* delayed appropriate therapy, *E. coli Escherichia coli*, *ESBL* extended-spectrum beta lactamase, *KPC K. pneumoniae* carbapenemase, *K. pneumoniae Klebsiella pneumoniae*, *NR* not reported, *UTI* urinary tract infection, *VPKP* VIM-1-producing *K. pneumoniae*

^a^Biliary tract, intestine, genital tract, bone/joint, meninges, respiratory tract, endocardium, skin/soft tissue, wound

^b^Peritoneum, pancreatobiliary tract, liver, lung, skin and soft tissue, bone, central nervous system

^c^Vascular catheter-related infection, intra-abdominal, bacteremia, skin and soft tissue

### Patient mortality by delayed appropriate therapy time frame

#### Overall delayed appropriate therapy

**Fig. 2:**
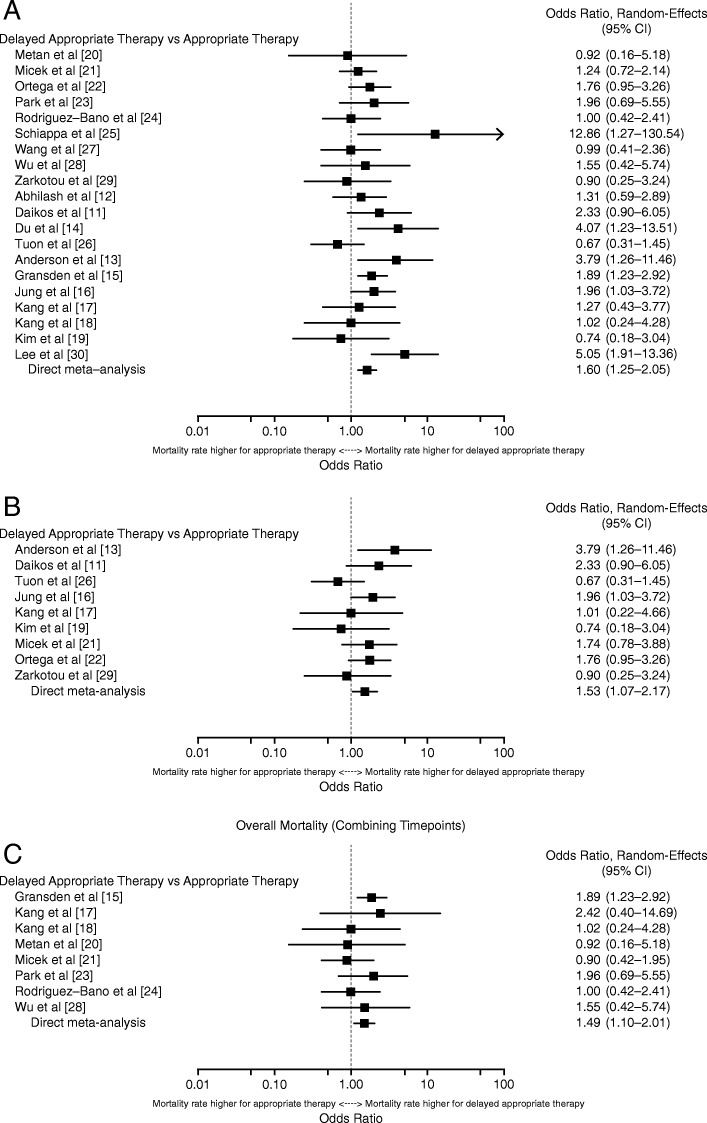
Overall mortality for all studies with any DAT > 24 h or DAT > C& SR of infections due to **(a)** *K. pneumoniae* or *E. coli,* **(b)** *K. pneumoniae* alone, and **(c)** *E. coli* alone

When all DAT results were combined for meta-analysis, infection due to *K. pneumoniae or E. coli* was associated with a significantly higher risk of overall mortality (OR, 1.60 [95% CI, 1.25–2.05]; Fig. 2a [11–30]). Heterogeneity was low (I^2^ = 32.4%, Qp = 0.130), with the most apparent outliers seen in Tuon et al. [26] on the low end (OR = 0.67) and Lee et al. [30] and Schiappa et al. [25] on the high end (OR = 5.05 and 12.86, respectively).

In the 9 studies reporting outcomes for patients with *K. pneumoniae* infections [11, 13, 16, 17, 19, 21, 22, 26, 29], the overall DAT was associated with a significantly higher risk of overall mortality (OR, 1.53 [95% CI, 1.07–2.17]; Fig. 2b [11, 13, 16, 17, 19, 21, 22, 26, 29]). With the exception of 3 studies [19, 26, 29], the proportion of patients who died was higher in those patient groups that received DAT compared with those receiving early appropriate antibiotics. Heterogeneity was low (I^2^ = 25.2%, Qp = 0.226), with the most extreme results being an OR of 0.67 on the lower end in Tuon et al. [26] and an OR of 3.79 on the high end in Anderson et al. [13].

Eight studies reported overall mortality data for patients with *E. coli* infection alone [15, 17, 18, 20, 21, 23, 24, 28]. In these studies, DAT was similarly associated with a significantly higher risk of overall mortality (OR, 1.49 [95% CI, 1.10–2.01]; Fig. 2c [15, 17, 18, 20, 21, 23, 24, 28]). There was no sign of heterogeneity in the results (I^2^ = 0.0%, Qp = 0.697). The proportion of patients who died was higher in those groups receiving DAT than in those who received appropriate therapy in 5 of the 8 studies (Table 1).

#### Delayed appropriate therapy > 48 hours or delayed appropriate therapy > C& SR, combined

**Fig. 3:**
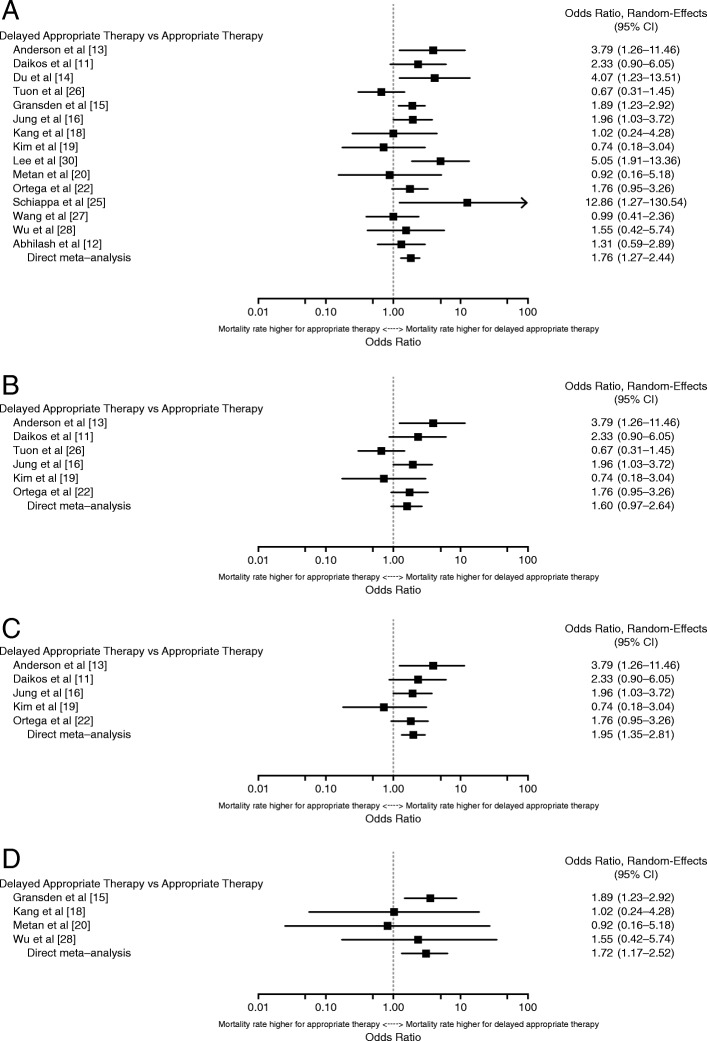
Overall mortality for all studies with a DAT > 48 h or DAT > C& SR for infections due to **(a)** *K. pneumoniae* or *E. coli*, **(b)** *K. pneumoniae* alone, **(c)** *K. pneumoniae* with Tuon et al. [26] outlier removed, and **(d)** *E. coli* alone

Meta-analysis results comparing overall mortality in patients with *K. pneumoniae or E. coli* infections who received DAT > 48 h or DAT > C& SR combined (*n* = 15) are shown in Fig. 3a [11–16, 18–20, 22, 25–28, 30]. There was a statistically significant increase in the rate of mortality associated with combined results from DAT > 48 h or DAT > C& SR (OR, 1.76 [95% CI, 1.27–2.44]). Heterogeneity was moderate (I^2^ = 45.8%, Qp = 0.070) and was driven by Tuon et al. [26] and Kim et al. [19] on the low end and Schiappa et al. [25] on the high end.

Three studies compared overall mortality in patients with infections due to *K. pneumoniae* who received DAT > 48 h [13, 19, 26], and another 3 studies reported results for DAT > C& SR [11, 16, 22], Fig. 3b [11, 13, 16, 19, 22, 26] depicts the quantitative synthesis of these data, resulting in an OR of 1.60 (95% CI, 0.97–2.64). Although the results suggest an increase in mortality associated with DAT > 48 h or DAT > C& SR compared with early appropriate therapy, the increase was not statistically significant. Moderate heterogeneity existed (I^2^ = 51.6%, Qp = 0.092) and was driven by the results in Tuon et al. [26], with an OR of 0.67. Tuon et al. was also 1 of 2 studies [19, 26] that showed a numeric trend of a higher rate of mortality in patients who received early appropriate therapy compared with those who received DAT > C& SR. When a sensitivity analysis was conducted with Tuon et al. removed, the results became statistically significant, as shown in Fig. 3c [11, 13, 16, 19, 22] (OR, 1.95 [95% CI, 1.35–2.81]), and there was no sign of heterogeneity (I^2^ = 0.0%, Qp = 0.487).

Four studies [15, 18, 20, 28] described mortality associated with DAT > 48 h (*n* = 3) or DAT > C& SR (*n* = 1) of *E. coli* infections. In these studies, DAT > 48 h or DAT > C& SR was associated with a significantly higher risk of overall mortality, with an OR of 1.72 (95% CI, 1.17–2.52; Fig. 3d [15, 18, 20, 28]). There was no sign of heterogeneity in the results (I^2^ = 0.0%, Qp = 0.748).

#### Delayed appropriate therapy > 24 hours

As shown in Table 2 [11–30], 5 studies described mortality associated with DAT > 24 h for either *K. pneumoniae* or *E. coli* infections [17, 21, 23, 24, 29]. There was a numeric trend suggesting a slight increase in mortality associated with DAT > 24 h (OR, 1.24 [95% CI, 0.85–1.80]; I^2^ = 0.0%, Qp = 0.876); however, these results were not statistically significant. For infections due to *K. pneumoniae* alone*,* there were no statistically significant differences for DAT > 24 h; 3 studies were included in the > 24-h analysis. Four studies described mortality associated with DAT > 24 h of *E. coli* infections alone. The OR of the meta-analysis was 1.19 (95% CI, 0.73–1.93), a result that was not statistically significant. There was no sign of heterogeneity in the results (I^2^ = 0, Qp > 0.20).

**Table 2:** Overall Mortality by DAT Definition for Infections Due to *Klebsiella pneumoniae* or *Escherichia coli*

Definition	*K. pneumoniae* or *E. coli* Infections		*K. pneumoniae* Infections Alone		*E. coli* Infections Alone	
	Studies, n [Ref]	OR (95% CI)	Studies, n [Ref]	OR (95% CI)	Studies, n [Ref]	OR (95% CI)
DAT > 24 h	5 [17, 21, 23, 24, 29]	1.24 (0.85–1.80)	3 [17, 21, 29]	1.37 (0.73–2.54)	4 [17, 21, 23, 24]	1.19 (0.73–1.93)
DAT > 48 h	4 [12, 15, 26, 28]	1.33 (0.84–2.12)	1 [26]	a	2 [15, 28]	1.86 (1.23–2.80)
DAT > 72 h	4 [13, 18, 19, 25]	2.12 (0.66–6.79)	2 [13, 19]	1.93 (0.25–12.48)	1 [25]	a
DAT > C& SR	7 [11, 14, 16, 20, 22, 27, 30]	2.06 (1.36–3.12)	3 [11, 16, 22]	1.93 (1.29–2.89)	1 [20]	a
All (Any DAT > 24 or DAT > C& SR)	20 [11–30]	1.60 (1.25–2.05)	9 [11, 13, 16, 17, 19, 21, 22, 26, 29]	1.53 (1.07–2.17)	8 [15, 17, 18, 20, 21, 23, 24, 28]	1.49 (1.10–2.01)

*C& SR* culture and susceptibility reporting, *DAT* delayed appropriate therapy, *E. coli Escherichia coli*, *K. pneumoniae Klebsiella pneumoniae*, *OR* odds ratio

^a^Analysis not possible owing to the identification of only 1 study with outcomes reported at the indicated time point for delay

#### Delayed appropriate therapy > 48 hours

Four studies described mortality associated with DAT > 48 h of either *K. pneumoniae* or *E. coli* infections (Table 2) [12, 15, 26, 28]. The meta-analyzed OR was 1.33 (95% CI, 0.84–2.12), and although the point estimate suggests an increase in mortality associated with DAT > 48 h, the result was not statistically significant. Heterogeneity was moderate (I^2^ = 36.4, Qp = 0.146) and was driven by the more extreme results in Tuon et al. [26] (OR = 0.67). For infections due to *K. pneumoniae* alone*,* there were insufficient data (*n* = 1) to conduct an analysis for DAT > 48 h. Two studies [15, 28] described mortality associated with DAT > 48 h of *E. coli* infections alone. DAT > 48 h was associated with a significantly higher risk of overall mortality (OR, 1.86 [95% CI, 1.23–2.80]). There was no sign of heterogeneity in the results (I^2^ = 0.0%, Qp = 0.778).

#### Delayed appropriate therapy > 72 hours

Four studies described mortality associated with DAT > 72 h of either *K. pneumoniae or E. coli i*nfections (Table 2) [13, 18, 19, 25]. The OR (2.12 [95% CI, 0.66–6.79]) of the meta-analysis was not statistically significant. Heterogeneity was moderate (I^2^ = 59.1%, Qp = 0.090) and was driven by the discrepancy of the results in Kim et al. [19] and Kang et al. [18] on the low end and Anderson et al. [13] and Schiappa et al. [25] on the high end. For infections due to *K. pneumoniae* alone*,* there were no statistically significant differences for DAT > 72 h; only 2 studies reported analyzable data to contribute to the > 72-h analysis. For infections due to *E. coli* alone*,* there were insufficient data (*n* = 1) to conduct an analysis for DAT > 72 h.

#### Delayed appropriate therapy > C& SR

Seven studies described mortality associated with DAT > C& SR of either *K. pneumoniae* or *E. coli* infections with an OR (95% CI) of 2.06 (1.36–3.12; Table 2) [11, 14, 16, 20, 22, 27, 30]. Heterogeneity was moderate (I^2^ = 33.5%, Qp = 0.211). For infections due to *K. pneumoniae* alone, DAT > C& SR was associated with a significantly higher risk of mortality (OR, 1.93 [95% CI, 1.29–2.89]). There was no sign of heterogeneity for this analysis (I^2^ = 0.0%, Qp = 0.887). For infections due to *E. coli* alone*,* there were insufficient data (n = 1) to conduct an analysis for DAT > C& SR.

## Discussion

There were several notable findings from this systematic literature review and meta-analysis that predominately (95%) included studies of patients with bloodstream infections due to *K. pneumoniae or E. coli*. First, the results suggest that overall, delays in appropriate therapy for patients with *K. pneumoniae* or *E. coli* infections result in higher mortality rates than when patients receive early appropriate therapy. On average, patients who receive any DAT (any delay exceeding 24 h after culture collection or C& SR) have up to 2-fold higher odds of death. This is consistent with the observed association between time to appropriate therapy and mortality for serious infections due to other pathogens [31]. Likewise, the results of this meta-analysis indicate that a delay in appropriate therapy that exceeds 48 h or lasts until after the time of C& SR is associated with an up to 2-fold higher risk of mortality. Taken together, the results for overall DAT suggest that even a delay of 24 to 48 h may negatively impact patient outcomes. Although not statistically significant, the ORs associated with DAT > C& SR only, which approximate a 48- to 72-h delay for reporting of culture and susceptibility data, were highly consistent with the ORs for a > 48- to > 72-h DAT. Finally, the results also suggest that the odds of mortality increased in a stepwise pattern as delays increased from > 24 to > 72 h. Although there were too few studies to draw definitive conclusions, it may be that the 24-h time point represents an inflection point beyond which a higher mortality risk is observed with increasing delays in initiating appropriate therapy. In general, the findings from this meta-analysis underscore the importance of timely initiation of appropriate therapy especially among patients with bloodstream infections due to *K. pneumoniae* or *E. coli*.

These findings have important implications for clinical practice, particularly highlighting the critical importance of using appropriate antibiotic therapy at initiation of treatment or “getting it right the first time.” Delivery of early, appropriate therapy is a fundamental pillar of antibiotic stewardship, and delivery of an appropriate drug during the course of an infection is one of the most important measures clinicians can take for their patients to ensure the highest probability of a successful outcome. In particular, the findings suggest that clinicians should be more aggressive with their empiric antibiotic choices, especially among patients with bloodstream infections due to *K. pneumoniae* or *E. coli*. As part of this proposed practice shift, it is of the upmost importance that therapy is “de-escalated” once culture and susceptibility data are available, when possible. Otherwise, prolonged overuse of broad spectrum antibiotic will exacerbate our growing resistance problems and will limit the future utility of current antibiotics. Because the increasing prevalence of antibiotic resistance increases the likelihood of a patient receiving inappropriate empirical therapy, our findings also highlight the need to develop tools to both identify patients at high risk for antibiotic resistance and facilitate rapid diagnosis of the underlying causative pathogen to ensure early appropriate therapy.

Several things should be noted when interpreting the findings. Nineteen of the 20 studies included in this meta-analysis were of patients with bloodstream infections due to *K. pneumoniae* or *E. coli.* It is unclear whether the results are applicable to other infection types. Study design limitations included those inherent to any meta-analysis, such as heterogeneity across studies that may originate from differences in study populations, including those that combined both hospital- and community- onset infections, different definitions of response, different definitions of in vitro susceptibility and appropriateness, and the lack of a multivariate control. Heterogeneity was noted in several of the meta-analyses. Interestingly, the studies driving the heterogeneity were those that assessed the association between DAT and infections due to ESBLs. The definition of appropriate therapy varied across these studies and likely contributed to the observed heterogeneity. For example, the study by Tuon and colleagues [26] only considered carbapenems as appropriate for the treatment of infections due to ESBLs and classified all other beta lactam therapies as inappropriate; and resulted in a lower rate for appropriate therapy. However, cefepime and piperacillin/tazobactam are active against most ESBLs and therefore may have been considered appropriate therapy in other studies.

The use of a key objective endpoint (mortality) across all studies helped to minimize any bias. Nonetheless, the presence of publication bias cannot be excluded. In addition, a targeted analysis taking into account all confounders was not possible, limiting the ability of this meta-analysis to definitively establish the presence of a causal relationship. Because most of the included articles were retrospective observational cohort studies that were not randomized with regard to DAT, possible treatment selection bias and bias in management decisions with respect to dosage adjustments could not be analyzed or accounted for in the present analysis. It was also difficult to determine a definitive cutpoint for time delay given the limited number of studies included in this analysis. It is possible that there are different cutpoints for different infection types or disease severities. Another potential limitation is that the published literature included here focused predominantly on adults with bloodstream infections; pediatric and other infection types might differ in terms of the impact of DAT. Additional studies in these other infection types are necessary to explore this question. For example, Kumar and colleagues [32] reported that appropriate antibiotic therapy within the first hour is associated with improved survival in patients with septic shock. A final limitation is that this analysis did not evaluate other outcomes, such as length of hospital stay and costs. Data are needed to evaluate how delays in therapy affect these outcomes and whether there are different time delay cut-points.

## Conclusions

In summary, results of this meta-analysis, which predominately (95%) included studies of patients with bloodstream infections due to *K. pneumoniae* or *E. coli,* indicate that delayed appropriate therapy increases the risk of mortality, especially when appropriate treatment is delayed by > 48 h. Given the results of this study documenting the adverse outcomes associated with delayed appropriate therapy, our findings suggest that there is a need to shift current treatment practices away from antibiotic escalation strategies that contribute to delayed appropriate therapy and toward early, relatively aggressive and comprehensive, antibiotic therapy, especially among patients with bloodstream infections due to *K. pneumoniae* or *E. coli*. As part of this proposed practice shift, it is critical that clinicians “streamline” therapy once culture and susceptibility data are available, when possible, to shorten the duration of time that patients unnecessarily receive broad spectrum antibiotics. Otherwise, prolonged overuse of broad spectrum antibiotics will exacerbate the growing resistance problem and will limit the future utility of current antibiotics. These findings also highlight the need for developing additional treatment options and strategies for infections in which therapeutic resistance is most likely to arise.

## Additional file

Additional file 1: Search Algorithms. (PDF 260 kb)

## Abbreviations

C& SR: Culture and susceptibility reporting
CAZ-R: Ceftazidime-resistant
CRE: Carbapenem-resistant Enterobacteriaceae
CTX-R: Cefotaxime resistant
DAT: Delayed appropriate therapy
*E. coli*: *Escherichia coli*
ESBL: Extended-spectrum beta lactamase
*K. pneumoniae*: *Klebsiella pneumoniae*
KPC: *K. pneumoniae* carbapenemase
NR: Not reported
OR: Odds ratio
PICOS-T: Participants, interventions, comparisons, outcomes, study design, and time period
PRISMA: Preferred Reporting Items for Systematic Reviews and Meta-Analyses
UTI: Urinary tract infection
VPKP: VIM-1-producing *Klebsiella pneumoniae*
