# Supplementary material for: A systematic review of the association between delayed appropriate therapy and mortality among patients hospitalized with infections due to Klebsiella pneumoniae or Escherichia coli: how long is too long?
Source: BMC Infect Dis. 2018 Dec 5;18:625. doi: 10.1186/s12879-018-3524-8 (PMC6280436; doi:10.1186/s12879-018-3524-8)
Supplement: Supplementary file 1 — Search Algorithms. (PDF 260 kb) [file 12879_2018_3524_MOESM1_ESM.pdf]

Additional file 1. Search Algorithms

MEDLINE (via PubMed)

Search Number	Search Algorithm	Hits
**#1**	“delayed appropriate therapy” OR “delayed therapy” OR “delayed antibiotic therapy” OR “delayed drug therapy” OR “delayed appropriate antibiotic therapy” OR “appropriate therapy” OR “appropriate drug therapy” OR “appropriate antibiotic therapy” OR “appropriate antimicrobial treatment” OR “appropriate antimicrobial treatment” OR “adequate treatment” OR “adequate therapy” OR “adequacy of treatment”	76,080
**#2**	“delayed inappropriate therapy” OR “delayed inappropriate antibiotic therapy” OR “inappropriate therapy” OR “inappropriate drug therapy” OR “inappropriate antibiotic therapy” OR “incorrect therapy” OR “incorrect antibiotic therapy” OR “incorrect treatment” OR “inadequate antimicrobial treatment” OR “inadequate antimicrobial therapy” OR time-to-therapy OR “time to therapy” OR “Ineffective empirical antibiotic therapy”	1517
**#3**	#1 OR #2	77,299
**#4**	“Gram-Negative Bacteria”[MeSH] OR (“Gram negative” AND (bacteria OR bacteremia OR “bloodstream infection” OR bacterium OR infection OR isolate OR culture OR pathogen)) OR “Gram-Negative Bacterial Infections”[MeSH] OR pseudomonas OR Klebsiella pneumoniae OR Enterobacteriaceae	806,273
**#5**	“Bloodstream infections” OR “Bacteremia” [MeSH] OR bacteremia OR “Intra-abdominal Infections” [MeSH] OR “complicated intra-abdominal infection” OR “intra-abdominal infection” OR “complicated intra-abdominal infection” OR “Urinary Tract Infections” [MeSH] OR “complicated urinary tract infection” OR “urinary tract infection” OR “Pneumonia, Bacterial” [MeSH] OR “bacterial pneumonia”	133,951
**#6**	#4 OR #5	906,189
**#7**	#3 AND #6	3533

Search Number	Search Algorithm	Hits
**#8**	Guideline [pt] OR “practice guideline”[pt] OR Letter [pt] OR editorial [pt] OR review[pt] OR news[pt] OR comment[pt] OR “case reports”[pt] OR “in vitro” [pt]	5,096,041
**#9**	Animals [mh] NOT humans [mh]	3,895,735
**#10**	#7 NOT (#8 OR #9)	1735
**#11**	#10 limited to English publications	1468
**#12**	**#11 NOT Child: birth–18 years; Infant: birth–23 months**	**975**

EMBASE

Search Number	Search Algorithm	Hits
**#1**	'delayed appropriate therapy' OR 'delayed therapy' OR 'delayed drug therapy' OR delayed NEAR/3 therapy OR (delayed NEAR/3 antibiotic AND therapy) OR appropriate NEAR/3 antibiotic OR appropriate NEAR/3 therapy OR 'appropriate therapy' OR 'appropriate drug therapy' OR 'appropriate antibiotic therapy' OR 'appropriate antimicrobial treatment' OR 'appropriate antimicrobial treatment' OR 'adequate treatment' OR 'adequate therapy' OR 'adequacy of treatment'	33,323
**#2**	'delayed inappropriate therapy' OR 'delayed therapy'/exp OR (delayed NEAR/3 antibiotic AND ('therapy'/exp OR therapy)) OR (inappropriate NEAR/3 antibiotic) OR (inappropriate NEAR/3 therapy) OR 'inappropriate therapy' OR 'inappropriate drug therapy' OR 'inappropriate antibiotic therapy' OR 'incorrect therapy' OR 'incorrect antibiotic therapy' OR 'incorrect treatment' OR 'inadequate antimicrobial treatment' OR 'inadequate antimicrobial therapy' OR time-to-therapy OR 'time to therapy' OR 'Ineffective empirical antibiotic therapy'	10,819
**#3**	#1 OR #2	37,304
**#4**	'Gram negative' OR ('Gram negative' AND (bacteria OR bacteremia OR "bloodstream infection bacterium" OR infection OR isolate OR culture OR pathogen)) OR 'Gram negative bacterium'/exp OR 'Gram negative sepsis'/exp OR 'Gram negative infection'/exp OR pseudomonas OR Klebsiella pneumoniae OR Enterobacteriaceae	532,479
**#5**	'Bloodstream infections' OR 'bacteremia'/exp OR bacteremia OR 'abdominal infection'/exp OR 'intra-abdominal infection' OR 'complicated intra-abdominal infection' OR 'urinary tract infection'/exp OR 'complicated urinary tract infection' OR 'urinary tract infection' OR 'bacterial pneumonia'/exp OR 'bacterial pneumonia'	119,856
**#6**	#4 OR #5	619,100
**#7**	#3 AND #6	4177

Search Number	Search Algorithm	Hits
**#8**	[editorial]/lim OR [erratum]/lim OR [letter]/lim OR [note]/lim OR [review]/lim OR ‘case report’/de OR ‘clinical protocol’/de OR ‘letter’:it OR ‘editorial’:it OR ‘review’:it	4,220,829
**#9**	#7 NOT #8	2734
**#10**	#9 AND [humans]/lim	2155
**#11**	#10 AND [English]/lim	2019
**#12**	**#11 limited to adults**	**1035**

Cochrane Databases

Search Number	Search Algorithm	Hits
**#1**	"delayed appropriate therapy" OR "delayed therapy" OR "delayed antibiotic therapy" OR "delayed drug therapy" OR "delayed appropriate antibiotic therapy" OR "appropriate therapy" OR "appropriate drug therapy" OR "appropriate antibiotic therapy" OR "appropriate antimicrobial treatment" OR "appropriate antimicrobial treatment" OR "adequate treatment" OR "adequate therapy" OR "adequacy of treatment"	682
**#2**	"delayed inappropriate therapy" OR "delayed inappropriate antibiotic therapy" OR "inappropriate therapy" OR "inappropriate drug therapy" OR "inappropriate antibiotic therapy" OR "incorrect therapy" OR "incorrect antibiotic therapy" OR "incorrect treatment" OR "inadequate antimicrobial treatment" OR "inadequate antimicrobial therapy" OR "time-to-therapy" OR "time to therapy" OR "Ineffective empirical antibiotic therapy"	71
**#3**	#1 OR #2	746
**#4**	("Gram negative" AND (bacteria OR bacteremia OR "bloodstream infection" OR bacterium OR infection OR isolate OR culture OR pathogen)) OR pseudomonas OR Klebsiella pneumoniae OR "Enterobacteriaceae"	3549
**#5**	MeSH descriptor: [Gram-Negative Bacteria] explode all trees	5648
**#6**	MeSH descriptor: [Gram-Negative Bacterial Infections] explode all trees	5765
**#7**	MeSH descriptor: [Bacteremia] explode all trees	749
**#8**	MeSH descriptor: [Intra-abdominal Infections] explode all trees	939
**#9**	MeSH descriptor: [Urinary Tract Infections] explode all trees	2065
**#10**	MeSH descriptor: [Pneumonia, Bacterial] explode all trees	683

Search Number	Search Algorithm	Hits
**#11**	“Bloodstream infections” OR “bacteremia” OR “complicated intra-abdominal infection” OR “intra-abdominal infection” OR “complicated intra-abdominal infection” OR “complicated urinary tract infection” OR “urinary tract infection” OR “bacterial pneumonia”	5597
**#12**	#4 OR #5 OR #6 OR #7 OR #8 OR #9 OR #10 OR #11	16,111
**#13**	**#3 AND #12**	**71**
